# An ontology for *Xenopus *anatomy and development

**DOI:** 10.1186/1471-213X-8-92

**Published:** 2008-09-25

**Authors:** Erik Segerdell, Jeff B Bowes, Nicolas Pollet, Peter D Vize

**Affiliations:** 1 Department of Biological Sciences, University of Calgary, Calgary, Alberta, Canada; 2 Department of Computer Science, University of Calgary, Calgary, Alberta, Canada; 3 Laboratoire Developpement et Evolution, CNRS UMR 8080, Université Paris-Sud, Orsay 91405, France; 4 Epigenomics Program, Genopole, Université Evry, Evry 91034, France

## Abstract

**Background:**

The frogs *Xenopus laevis *and *Xenopus (Silurana) tropicalis *are model systems that have produced a wealth of genetic, genomic, and developmental information. Xenbase is a model organism database that provides centralized access to this information, including gene function data from high-throughput screens and the scientific literature. A controlled, structured vocabulary for *Xenopus *anatomy and development is essential for organizing these data.

**Results:**

We have constructed a *Xenopus *anatomical ontology that represents the lineage of tissues and the timing of their development. We have classified many anatomical features in a common framework that has been adopted by several model organism database communities. The ontology is available for download at the Open Biomedical Ontologies Foundry .

**Conclusion:**

The *Xenopus *Anatomical Ontology will be used to annotate *Xenopus *gene expression patterns and mutant and morphant phenotypes. Its robust developmental map will enable powerful database searches and data analyses. We encourage community recommendations for updates and improvements to the ontology.

## Background

The African clawed frog, *Xenopus laevis*, is a widely used model organism in developmental biology and the related species *Xenopus (Silurana) tropicalis *has emerged as an important model for genetics. The quickly developing embryos of *Xenopus*, which form a full set of differentiated tissues within days of fertilization, have been described in fine detail [1] and are highly amenable to analysis of embryonic gene function. High throughput screens of gene expression patterns by whole mount *in situ *hybridization in *X. laevis *[2,3] and morpholino-based gene-knockdown experiments [4,5] have generated a vast set of gene function data. These data are crucial to ongoing *Xenopus *research efforts and they complement the gene expression and gene function information that is available for other model systems. Consequentially a need has emerged for a centralized database of *Xenopus *information, and with it, in order to organize tissue-specific data, a formalized specification of knowledge about *Xenopus *anatomy throughout the organism's development. Biomedical ontologies offer distinct advantages for annotating and disseminating biological data, representing areas of knowledge such as gene function, genetic sequence features and anatomy as structured, controlled vocabularies [6] and giving researchers and informaticians the ability to query and communicate across biological and human disease databases. We began work on a new anatomical ontology when we launched Xenbase as the model organism database for *Xenopus *[7] and started to organize diverse types of developmental and genomic data.

For biomedical ontologies in general to successfully address the challenges of data integration, biologists, clinicians and computer scientists have come together to develop design principles that promote formal rigor and high quality in ontology development and launched a collaborative experiment in driving the evolution of ontologies, the Open Biomedical Ontologies (OBO) Foundry [6]. The foundry serves as a central repository for existing ontologies and encourages the application of the scientific method to ontology development in order to bring about their improvement. Anatomical ontologies [8] are a major subset of the open, orthogonal bio-ontologies that are listed at the foundry's website [9]. The *Xenopus *Anatomical Ontology's development has occurred at a time of active refinement of OBO's best practices and principles and the emergence of a Common Anatomy Reference Ontology (CARO), which provides a template for building model organism and multi-species anatomical ontologies and is intended to foster interoperability between existing ones [10]. Furthermore, an effort to standardize amphibian anatomical nomenclature has been launched under the auspices of the Amphibian Anatomical Ontology [11], presenting an additional opportunity for collaboration that can lead to a *Xenopus *resource of maximal relevance and utility.

In support of the need to integrate the wealth of *Xenopus *genetic, genomic, and developmental information both within a central community database and with external model organism and biomedical resources, we have constructed an ontology of *Xenopus *anatomical structures and tissues. It contains a map of development from embryo to adult in several major anatomical systems, the most robust such map in a vertebrate model system. We report here its availability at Xenbase and the OBO Foundry and we describe our ongoing effort to create a resource that will be valuable to the *Xenopus *research community and that will contribute to the evolution of bio-ontologies as essential tools for representing biological knowledge.

## Results

### The *Xenopus *Anatomical Ontology

In the *Xenopus *Anatomical Ontology (XAO) we have described *X. laevis *anatomy as a controlled, structured vocabulary of the set of structures and tissues that exists throughout development. Items in the XAO, which we here call "types" as recommended by Smith et al. [12], exist in a graphical structure where they may have multiple "parents" or multiple "children" and are connected by relationships. A given tissue may be asserted to be *part_of *another tissue and it may be classified as a subtype of another tissue, with which it is said to have an "*is_a*" relationship. We have arranged the XAO's topmost nodes using CARO, the Common Anatomy Reference Ontology, as a template, and CARO's relationships and definitions [10] have been adopted for these structure types. As an essential framework for representing developmental events, we have incorporated the complete Nieuwkoop and Faber staging series into the ontology. The 66 stage types in this sub-ontology and their definitions (including time since fertilization and external staging criteria) have been compiled from the Normal Table of *Xenopus *development [1]. Individual NF stages have been grouped into six superstages: cleavage, blastula, gastrula, neurula, tailbud and tadpole. The XAO represents the transformation, fission, and fusion of structures and tissues throughout embryonic and larval development with a network of *develops_from *relationships between them.

Each type in the ontology has been automatically assigned a unique identifier that has two components, an approved abbreviation for the ontology (XAO) and a serial number associated with only that entity. An ID remains stable even if the agreed-upon nomenclature for a structure or tissue changes. If a type is later designated as "obsolete", its ID will not be re-used for a new term. Synonyms, including plural forms, have also been assigned to several structures and tissues.

Stage types have been organized according to recommendations by the CARO working group [10]. Their relative timing is indicated by *preceded_by *relationships (for instance, "NF stage 7" is *preceded_by *"NF stage 6.5"). The series of stages has given us a basis for indicating the time during which an anatomical structure or tissue is known to exist by creating start- and end-stage relationships, e.g. "pronephric kidney" *starts_during *"NF stage 29/30" and *ends_during *"NF stage 55". We have taken each anatomical type's other relationships into account when curating stage range information, adopting rules first developed for the Zebrafish Anatomical Ontology [13]: types with *is_a *and *part_of *relationships follow the rule that a child must exist within the range of its parent and types with *develops_from *relationships follow the rule that a child's range must overlap or abut the range of its parent. Thus in the timeline of embryonic and larval development, for "pronephric kidney" to have a valid *develops_from *relationship to its parent "pronephric mesenchyme", it must be specified as appearing no later than stage 31 (late tailbud), the NF stage immediately following the latter's end stage; and "glomus", being *part_of *"pronephric kidney", must not be specified to exist outside of the stage 29/30 to 55 range.

We have written type definitions that adhere to the OBO Foundry's recommended "genus-differentia" format [6]; e.g., "cell" (XAO: 0003012) is defined as an "anatomical structure" (the "genus") that has as its parts "a maximally connected cell compartment surrounded by a plasma membrane" (the "differentia", or distinguishing property or set of properties that sets off this entity from other anatomical structures), and "pigment cell" (XAO: 0003014) is a "cell (genus) that contains coloring matter (differentia)".

**Figure 1:**
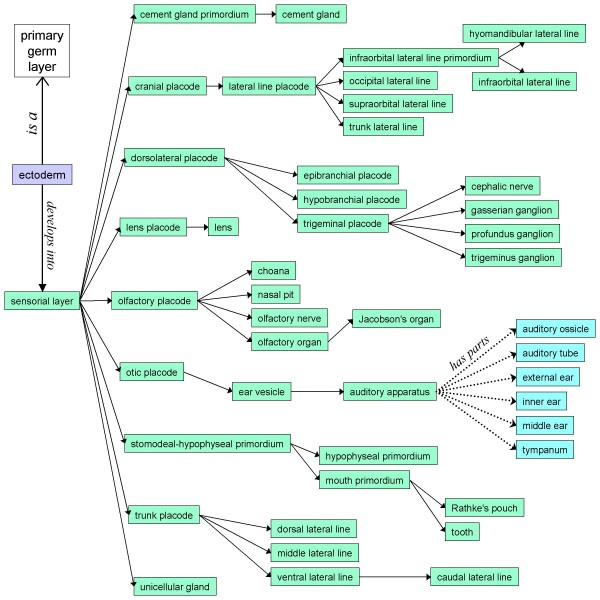
**A portion of the *Xenopus *Anatomical Ontology's developmental map**. Shown are the anatomical features to which the sensorial layer of the ectoderm gives rise. Types in green boxes have *develops_from *relationships to their parents. Also shown here are examples of the other two anatomical relationships that are integral to the XAO: ectoderm *is_a *primary germ layer; and the auditory ossicle, etc., are *parts_of *the auditory apparatus. The *develops_from *relationships are transitive: given that the lens develops from the lens placode and the lens placode develops from the sensorial layer, it is implied that the lens develops from the sensorial layer (and more generally from ectoderm). Transitivity operates over *is_a *relationships as well, e.g. the lens may be inferred to develop from a primary germ layer.

The XAO contains 87 stage types, including superstages, and 626 anatomical types. *Xenopus *development is represented by 308 *develops_from *relationships. "Embryo" (XAO:0000113) and "anatomical structure" (XAO:0003000) are high-level nodes of the anatomical ontology and provide the starting points for a developmental map and for a structural classification scheme, respectively. The map of *Xenopus *development is organized by primary germ layer. The sensorial layer of ectoderm provides an example of the lineage information that can be found (Figure 1); in the XAO several lineages have a depth of four or five nodes. In the "anatomical structure" classification scheme, many tissues have been organized under five "organism subdivisions": the head, trunk, tail, limbs and surface structures. In addition, tissues have been classified in eleven anatomical systems (e.g., the nervous system). The lateral line system, for example, is represented as a part of the nervous system, as a surface structure, and as a derivative of ectoderm, allowing it to be found either by structural subdivision or by lineage.

Xenbase serves as the primary repository and center of development of the XAO [7]. The XAO file is freely available at Xenbase's FTP site [14] and is deposited in the OBO Foundry [15].

### Ongoing XAO development

Given time and resource constraints, it is unrealistic to incorporate the entire vast body of knowledge about *Xenopus *anatomy and development, and we assess community input, annotators' needs, and our commitment to meeting good ontology development principles as we prioritize those specific areas of the ontology requiring further curation. Feedback from annotators using the XAO for the EuReGene Gene Expression Database [16], for example, identified embryonic tissue types in need of start- and end-stage information, and we expect that Xenbase's in-house curation of expression data will lead to the addition of new terms and more stage data and definitions to the ontology. Furthermore, some types have only *develops_from *relationships to other types and need additionally to be classified as subtypes or parts of other structures or tissues. Ideally, every type in the ontology should be assigned one *is_a *relationship to a supertype, providing the ontology with a clean classification structure and a framework for logical "genus-differentia" type definitions [10]. Alignment of the topmost levels of the XAO with CARO provides a basis for achieving this goal: for example, all cell types existing in the ontology have been classified under "cell" (XAO:0003012, CARO:0000013) with several definitions borrowed from the Cell Ontology [17]. We plan to collaborate with CARO developers to finish these assignments and to classify structures in additional categories such as "compound organ" and "simple organ" and a variety of tissue types (e.g., epithelium, mesenchyme).

XAO development will also benefit from our collaboration with Amphibian Anatomical Ontology developers and our participation in AAO workshops and ontology curation jamborees. Several XAO types, especially skeletal structures, have names and definitions that are based on the AAO. These types contain cross-references to the amphibian ontology. Further improvement of the skeletal system and the addition of detailed musculature system information to the XAO are near-term projects that will be facilitated by the relationship between our two groups.

## Discussion

We have built a *Xenopus *ontology containing a developmental map of large scope and have populated it with several hundred anatomical features. The ontology's structure permits computers to make inferences from the ontology that lead to robust database search capabilities [8]. The planned implementation of the XAO in support of gene expression data will enable Xenbase users to perform queries that are more powerful than simple text matches. The relationships between types in the ontology will produce deeper and more meaningful results: for instance, a search for genes expressed in "eye" can return data records annotated with "retina" (explicitly defined in the ontology as *part_of *the "eye"), even in cases where "eye" does not literally appear anywhere in a record. Users will be able to take advantage of *develops_from *relationships as well; for example, one might retrieve data for genes expressed in any derivative of "mesoderm" in a single search operation. The existence of unique identifiers for each type will ensure data integrity and database interoperability [8], and the ontology's support of synonyms should result in successful searches regardless of one's preferred anatomical nomenclature. Furthermore, constructing definitions by recommended "genus-differentia" rules has made the ontology consistent internally as well as consistent with other biomedical ontologies whose developers have committed to this practice.

The *develops_from *map has the potential to be powerfully applied in microarray analysis, for which *Xenopus *embryos present an ideal model of genome-wide expression analysis in early vertebrate development [18]. Once gene expression is mapped to specific tissues, the developmental map will allow expression to be linked not only to tissues, but also to points in time during embryogenesis; this would be a major breakthrough for investigating genes involved in temporal and inductive events in *Xenopus *development.

Anatomical ontologies have become increasingly vital for describing mutant, morphant, and disease phenotypes in a machine-processable format, with anatomical types being one component of a bipartite syntax called Entity-Quality (EQ) [19]. An ontology of phenotypic qualities (PATO) [6] can be used to describe how an observable entity in any species (an anatomical structure or tissue, biological process, molecular function, or cell type or component) differs from its wild-type phenotype; for example, a small eye phenotype in a *Xenopus *mutant would be described as E = "eye" (XAO:0000179) and Q = "decreased size" (PATO:0000587). Groups of EQ annotations and associated genotype information can foster cross-species analysis of vertebrate gene function and can constitute a shared method of relating phenotypes to human disease. This system is now used to annotate phenotypes in zebrafish and other model species and will be the standard for future phenotype support in Xenbase.

**Figure 2:**
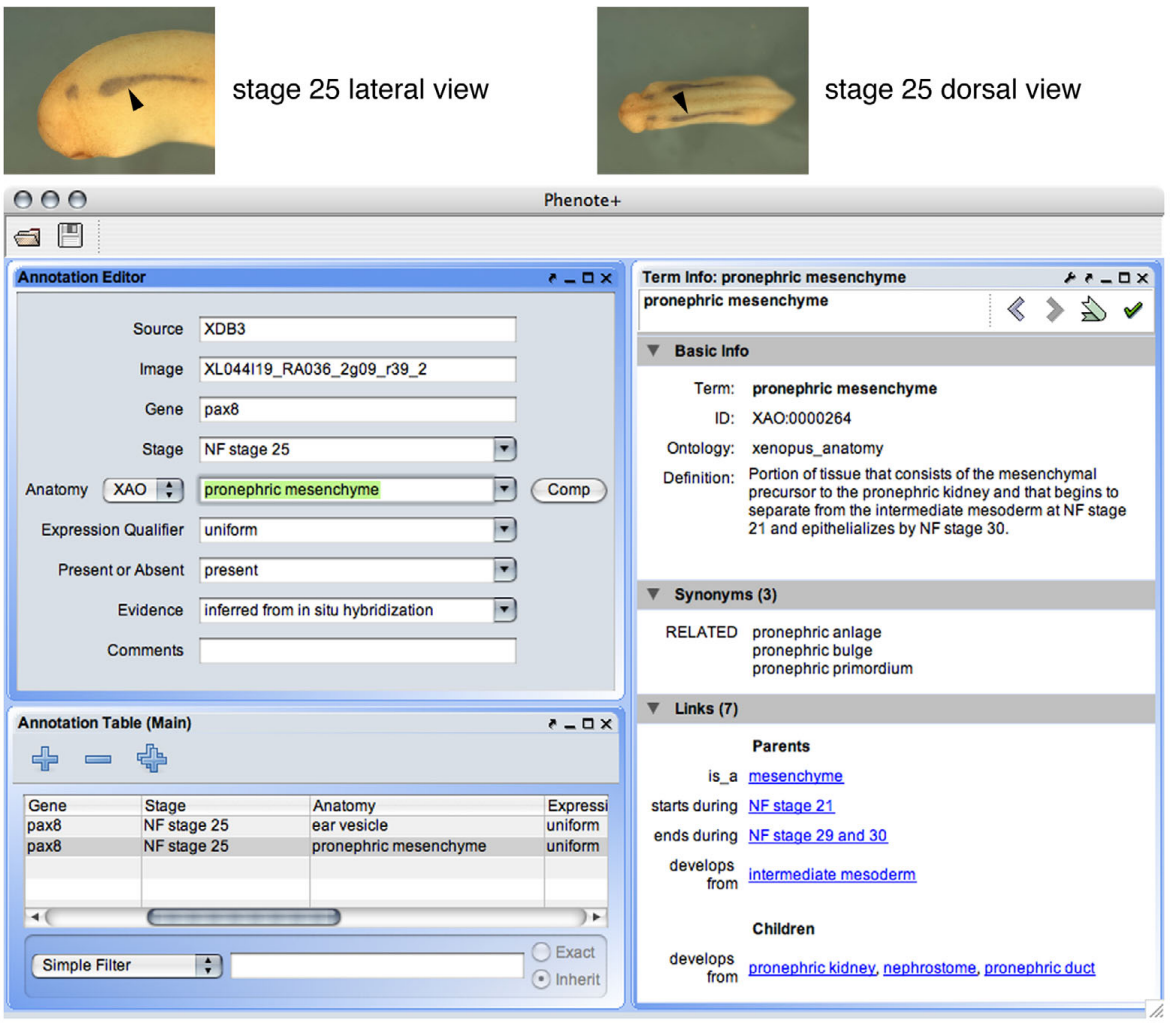
**Annotation of *X. laevis *gene expression in the Phenote tool with the XAO**. Images of a Nieuwkoop-Faber stage 25 embryo from the XDB3 *in situ *gene expression database (top) show pax8 expression in the developing pronephros (arrowheads), information that can easily be captured with the ontology-aware Phenote application. The XAO provides a controlled vocabulary for recording stage and anatomical information in the Phenote interface (drop-down menus, at left), while a separate ontology is used for assertions of evidence; data is recorded in a spreadsheet-like panel (bottom-left). The term viewer at right displays information about the current anatomy selection ("pronephric mesenchyme") including its unique XAO identifier, synonyms and definition, relationships to other types in the ontology, and the stage range during which it exists during development. Related types are hyperlinked to give annotators the ability to rapidly browse the ontology for contextual information or to seek a more appropriate item with which to populate an annotation.

A well-designed ontology enables the adoption of annotation tools that are more powerful and encourage more accurate, precise annotations than interfaces that contain only simple pick-lists of vocabulary items or free-text fields. Phenote [20] is one such tool, developed as an open source Java application that supports standard biomedical ontologies. Although it was developed originally for biomedical phenotype annotation, Phenote can be configured for any kind of data on which ontology-based annotations may be applied, such as gene expression (Figure 2). Upon launch, the application checks for updates to user-specified ontologies. During data entry, users can easily traverse an ontology with a webpage-like interface and find metadata for relevant types including definitions, related types, and stage ranges. Types can be found by entering synonyms and even parts of a definition. Such quick access to contextual information promotes consistent use of a shared vocabulary by different curators or disparate groups of annotators, reducing guesswork, inefficiency and errors that may result from having to choose from a simple list of terms.

## Conclusion

We have developed the *Xenopus *Anatomical Ontology in order to formalize the rich legacy of knowledge about the anatomy and embryonic and larval development of a widely used model vertebrate, and by making this resource easily accessible with common open-source bioinformatics tools we believe that it will be useful to biologists. By working closely with a committed and growing community of biomedical ontology developers and by providing feedback to improve Phenote, we have also tried to ensure that it will successfully fulfil its role in describing a massive amount of *Xenopus *gene function data. Its large developmental map and links between stages and tissues will enable powerful database searches and data analyses.

Community feedback will be invaluable for the *Xenopus *Anatomical Ontology's success as a robust bioinformatics resource. We encourage *Xenopus *biologists, anatomists and those with expertise in particular sets of anatomical structures to send comments and recommendations to the lead curator at xenbase@ucalgary.ca.

## Methods

We built the XAO in the open source graphical ontology editor OBO-Edit [21] and we continue to curate the ontology with the most recent official release of the program. The ontology is available as OBO and Web Ontology Language (OWL) format files and is maintained in a CVS repository at SourceForge [22]. In Xenbase the XAO is represented as a directed acyclic graph (DAG) implemented with relational data tables. Automatic loaders are used to synchronize these tables with updates to the OBO file.

XAO types, synonyms, definitions, and information about developmental events have been accumulated from textbooks and journals (especially Nieuwkoop and Faber [1]; also for example [23,24]), online biomedical resources (e.g. [25]), and our own expertise. Top-level anatomical structure types and their definitions are based on the topmost nodes of the Common Anatomy Reference Ontology.

## Authors' contributions

NP generated the first draft of the ontology. ES added additional terms, organized the ontology to be CARO compliant and drafted the manuscript. JBB provided guidance and organized implementation of the XAO in Xenbase. PDV built the *develops_from *map and added additional terms. All authors read and approved the final manuscript.
